# Mechanical properties of friction induced nanocrystalline pearlitic steel

**DOI:** 10.1038/s41598-022-16848-2

**Published:** 2022-07-22

**Authors:** B. Medina-Clavijo, J. Rafael-Velayarce, E. Modin, M. Saez-de-Buruaga, D. Soler, C. Motz, P. J. Arrazola, A. Chuvilin

**Affiliations:** 1 Electron-Microscopy Laboratory, CIC NanoGUNE BRTA, 20018 Donostia, Spain; 2 Department of Material Science and Technology, Saarland University, 66041 Saarbrücken, Germany; 3 Faculty of Engineering, Mondragon Unibertsitatea, 20500 Arrasate-Mondragon, Spain; 4 Ikerbasque, Basque Foundation for Science, 48013 Bilbao, Spain; 5 Present Address: European Organization for Nuclear Research (CERN), 1211 Geneva 23, Switzerland

**Keywords:** Materials for devices, Nanoscale materials, Structural materials, Techniques and instrumentation

## Abstract

Nanocrystalline structured variants of commercially available alloys have shown potential for boosting the mechanical properties of these materials, leading to a reduction in waste and thereby retaining feasible supply chains. One approach towards achieving these nanostructures resides in frictional treatments on manufactured parts, leading to differential refinement of the surface structure as compared to the bulk material. In this work the machining method is considered to be a testing platform for the formation and study of frictional nanostructured steel, assembly of which is stabilized by fast cooling of the produced chip. Analysis of the mechanical properties has shown extraordinary results at the surface, over 2000 MPa of strength on AISI1045 steel, more than three times the strength of the base material, demonstrating at the same time a reduction of 15% in the elastic modulus. The microscopic analysis suggests a reassembly of the elements in a new lattice of carbon supersaturated nano-ferrite.

## Introduction

Nanostructured metallic materials typically have very different mechanical properties as compared to their coarse-grained counterparts. As a consequence of the reduction of mobility of linear defects (dislocations), confined between close grain boundaries (GBs), the hardness and ultimate strength of nanostructured metals is very high, which might provide certain technological benefits. Traditional methods for fabricating nanostructured metals have been classified as bottom-up and top-down^1^. Bottom-up refers to the assembly of the material by agglomeration of atoms or molecules (as a rule, in non-equilibrium conditions), such as processes based on vapor deposition or fast solidification. The top-down approach on the other hand, consists of refining existing coarse structure primarily by severe plastic deformation^2^ (SPD). SPD has been implemented in several techniques for the refinement of a bulk material, e.g., high pressure torsion^3^ (HPT) or equal channel angular extrusion^4^ (ECAE).

SPD techniques modify the whole workpiece, thus providing a new bulk material with unique properties defined by nanocrystallinity. The other class of techniques, which are substantially less studied and developed, involve only surface modification. In many practical applications this method may even be beneficial for the piece’s consumer properties. This can be achieved through modification of a surface by friction with a tool in a process similar to friction welding or high-speed cutting.

The formation of surface layers with sub-micrometer grains has been reported in machining and tribological experiments^5,6^, however one can expect that the formation of these structures corresponds to different conditions compared to nano-structuring of the entire bulk material. For instance, the characteristic strain rate in the friction area during machining covers values between 10^3^ and 10^5^ s^−1^^7^, where the strain-induced heating has a major impact. Temperatures over 0.5 and up to 0.8 of the melting temperature are common in machining, activating processes like dynamic recrystallization^8^. Investigations on the chip friction surface moving over and away from the cutting tool suggests not only a strong refinement^6,9,10^ but also substantial transformations in the distribution of the elements of the alloy. The grain size observed in the friction surface of 1045 AISI steel remained below 100 nm in the top layer. Crystal size refinement was accompanied by a strong redistribution of the alloying elements from the original pearlitic structure, and by a reduction of the residual stress as a consequence of a process of dynamic recrystallization^11^. While efficient procedures to study the intermediate events during fast friction induced transformations of the surface of metals are emerging^12–14^, current setups to study machining represent a developmental platform to learn about conditions and structures of these nano-structured materials, which are efficiently preserved by the ultra-fast cooling happening in chips after cutting^8^.

Properties of bulk nanostructured metals are well known and are typically characterized by an increase of the strength and a detrimental decrease in thermal stability, common for both bottom-up and top-down fabrication methods^15^. At the same time the mechanical properties of the final and intermediate stages of thin layers of friction-induced nanomaterials (FIN) have, to the knowledge of the authors, only recently become a matter of deep analysis, hampered by the typical thickness (of some microns) of the layer affected by friction. Pillar testing experiments have shown that these materials develop different collapsing tendencies under ultimate compression and a high material strength, hence they possess fundamentally different deformation mechanics^16^. A very promising finding of Zhou et al. suggests that in terms of thermal stability, these structures demonstrate a strong deviation from known nanomaterials^17^, i.e., FINs depict an overwhelming thermal stability, comparable to supramicron structured alloys. These structures were obtained by surface sliding in cryogenic conditions, leading to a fast local self-heating due to the large strain rate and consequent fast cooling. This opens further applications of nanomaterials for industrial applications, since the long-term stability could be solved with the proper mechanical refining at the surface. Similar grain sizes can be readily obtained in the chip after fast machining of the steel, where the material is heated up to 0.6 of the melting temperature (Tm) by enormous strain rate and cooled down in timeframe of milliseconds^8^. Thus, this process can provide a benchmark material to test the properties of potential future nano-structured alloys.

In this work we present a study of the FIN obtained from 1045 AISI steel in high-speed cutting process. Morphological, structural, chemical and mechanical testing has been performed on the samples. A set of pillar compressions along with the crystal size gradient in the chip surface has permitted the evaluation of bulk-equivalent ultimate strength at different grain sizes, down to sub-100 nm crystalline material.

## Results

**Figure 1:**
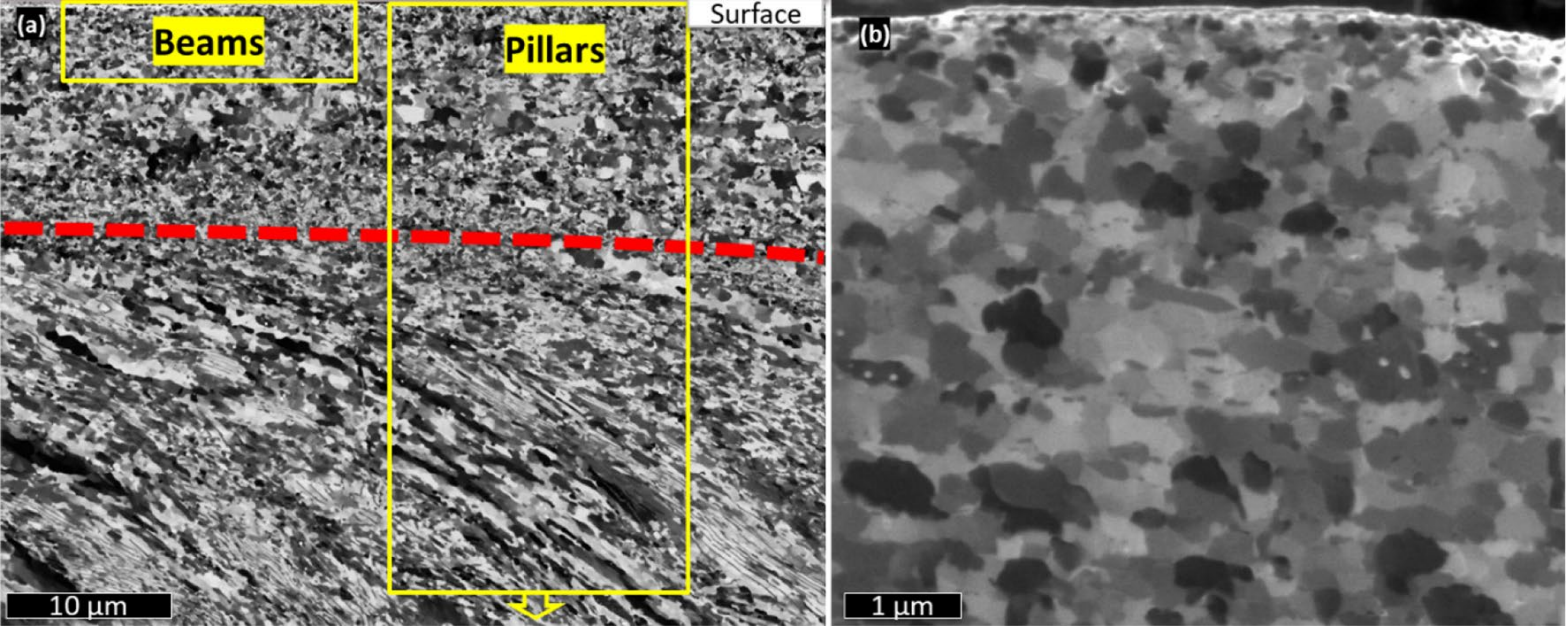
Ion image of the chip cross-section machined at 200 m/min. (**a**) Red dashed line is an estimation of PSZ-SSZ boundary. (**b**) Detail of the SSZ by ion scanning of chip cross-section when machining at 200 m/min. Crystals tend to equiaxiality and the diameter is consistently in the sub-micrometer range.

After machining, the material in the chip shows a strong structural refinement. Figure 1 shows a channeling contrast focused ion beam (FIB)^18^ image of the cross-section of the chip. The surface that was in contact with the tool is at the top of the image, the red dashed line separates two areas with distinctly different structures, a consequence of different recrystallisation conditions. During machining most of the shearing leading to generation of the chip occurs well ahead of the tool, in the so called primary shear zone (PSZ^7^), leading to the microstructure under the dashed line, where the texture reveals a shear direction with a parallel distribution of the long axis of the crystals. Closer to the surface the material is sheared by sliding along the cutting tool (the secondary shear zone, SSZ^7^). In this zone the effects of friction are more pronounced resulting in the very small crystallites and no signs of a particular shear direction. This last structure is typical for a material that has undergone dynamic recrystallization (DRX) in FIN samples and is known as a “white layer” in corresponding literature^19^. Both sheared and recrystallized structures have been mechanically tested in the areas indicated by yellow boxes. The pillar compression tests covered both recrystallized and sheared material, while beam bending was only performed on sub-100 nm grained material at the very surface due to the necessity for side access.

**Figure 2:**
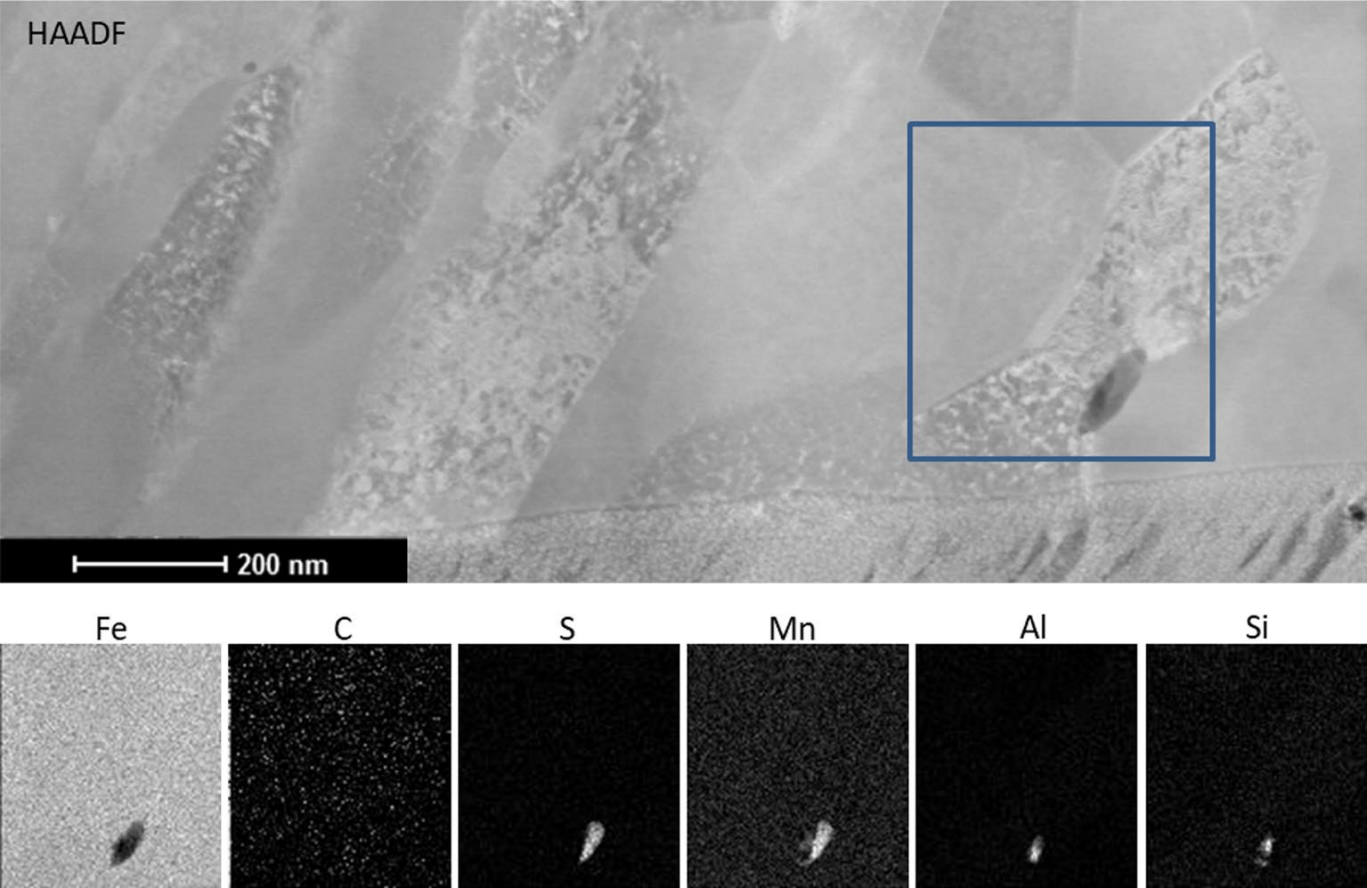
STEM-HAADF image of the DRX area (top). EDX chemical maps (bottom row) of the square in the HAADF image depict the distribution of Fe, C, S, Mn, Al and Si. C is equally distributed and does not show excess neither in the inclusion nor in the grain boundaries. Elements typical for impurities in commercial steels constitute the inclusion.

In Fig. 1a a certain gradient of grain sizes can be observed, from sub-micron at the boundary of the DRX layer and down to 80 nm in the top surface (Fig. 1b), which compared to the original material structure denotes a two orders of magnitude reduction in grain size. Moreover, in contrast to the original material, which has pearlite colonies in 75% of the volume, the recrystallized material presents a lack of cementite lamellae (see Fig. 1b and further discussion on Fig. 2). FEM simulations with similar conditions suggested that the recrystallized area develops strains of over 300% (see details in Methods), introducing energy to initiate a DRX process.

The DRX area has been analyzed by scanning transmission electron microscopy (STEM). Figure 2 (top) shows a STEM image of a few grains from the very top of the DRX layer (protective Pt layer used in FIB milling is visible at the bottom of the image). A sole inclusion which might be attributed to the residual cementite is in fact constituted of sulfur, manganese, silicon and aluminium as is evident from EDX maps on Fig. 2 (bottom). The chemical analysis revealed that carbon is distributed homogeneously without segregation in the grain boundaries or as cementite grains. Some of the typical impurity elements of steel persist as agglomerates.

**Figure 3:**
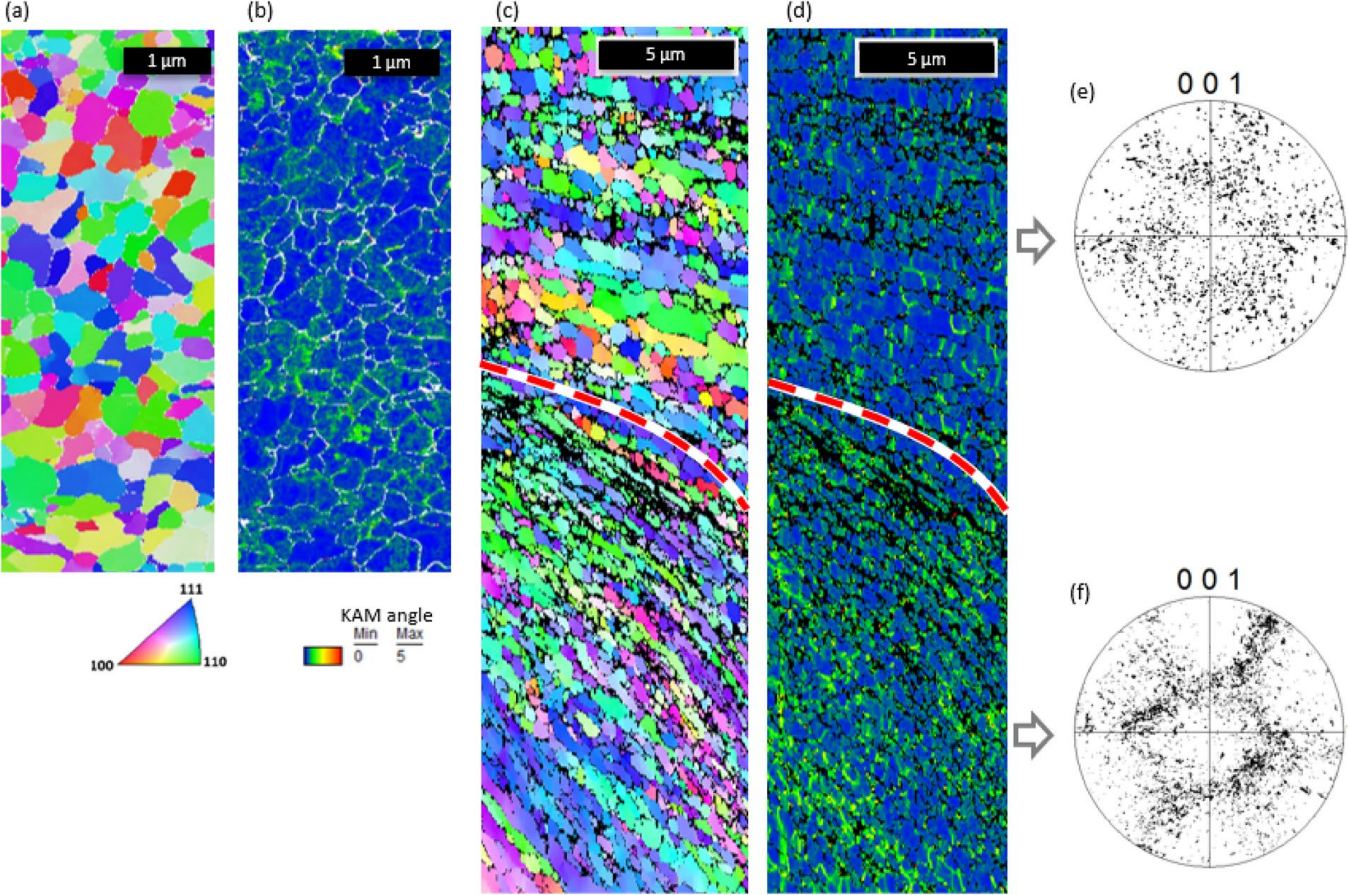
Crystallographic orientation by EBSD on frictioning surface (top corresponds to chip-tool contact surface) on chips cut at 200 m/min. (**a**) Inverse pole figure (IPF) colored in the proximity of the surface and (**b**) corresponding Kernel average misorientation (KAM) map. (**c**) IPF and (**d**) KAM of the whole area affected by friction. (**e**) and (**f**) are pole figures (PF) of the area inside and outside of the DRX layer respectively.

The crystallographic structure of the recrystallized and sheared area has been further analyzed by (transmission) electron back scatter diffraction ((t)-EBSD) in a scanning electron microscope (SEM). Figure 3a shows a t-EBSD map of the material at the very surface of the DRX layer (depth increases from top to the bottom of the map), Fig. 3b shows the corresponding kernel average misorientation (KAM) map. EBSD data in Fig. 3c,d provide a broader view of the DRX layer on top and sheared region at the bottom. The sheared layer shows a structure with a prominent shear deformation, where grains/sub-grains with similar orientation are aligned in lamellae at an oblique angle to the top surface. In contrast, the crystals in the DRX layer have an equiaxial shape without prominent signs of shear. Pole figures (Fig. 3e,f) show a significantly more prominent crystal texture of the sheared layer as compared to the DRX. The KAM map is an illustrative indicator of the density of geometrically necessary dislocations^20^. Dislocation density is visually substantially lower in the DRX layer (Fig. 3b,d) confirming structural reorganization (internal strain release) by re-crystallization.

**Figure 4:**
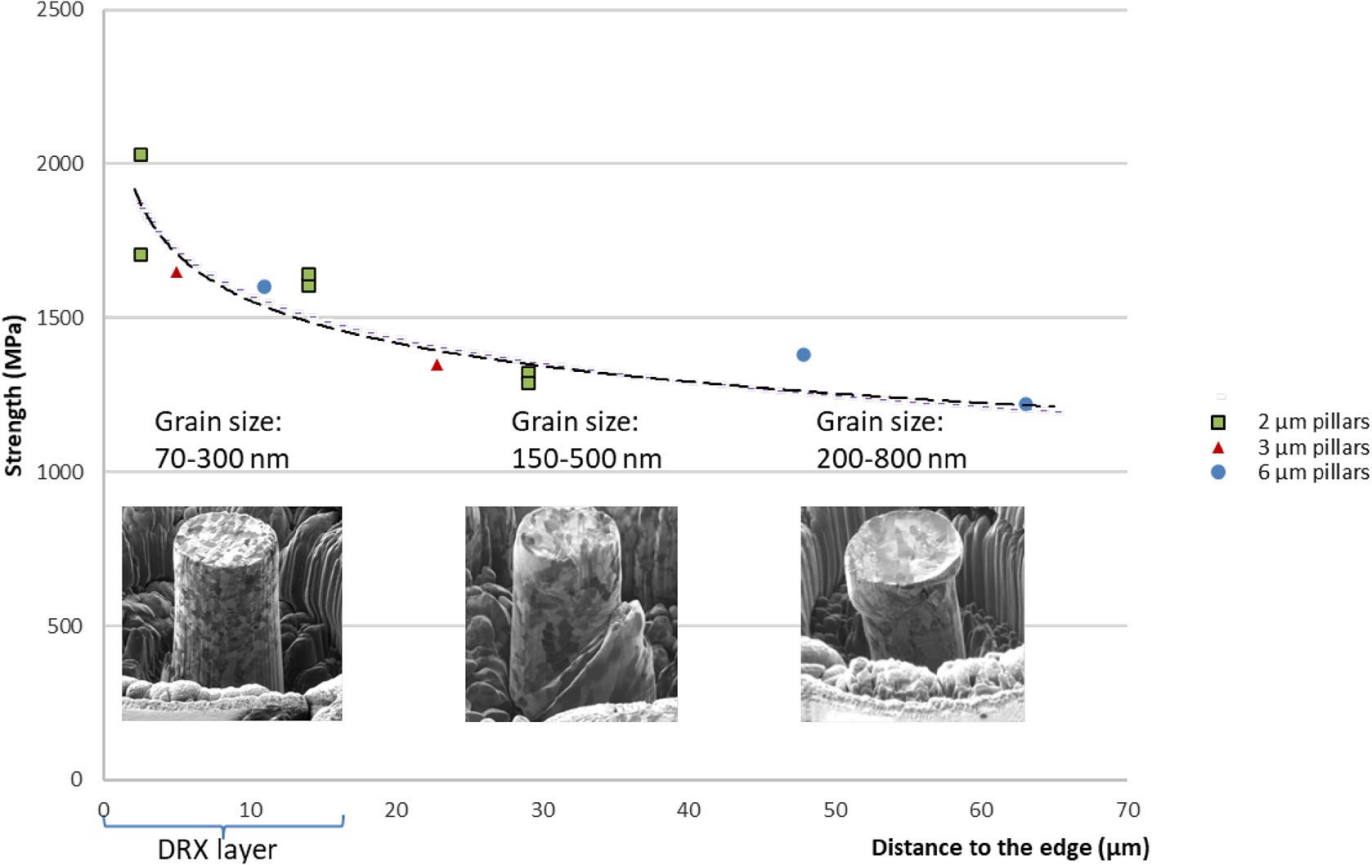
Strength values obtained by pillar compression tests of the pillars of 2, 3 and 6 µm of diameter milled on a cross-section at different depth into the material. Pillar diameter does not have notable influence, however pillar closer to the edge show larger strength.

**Figure 5:**
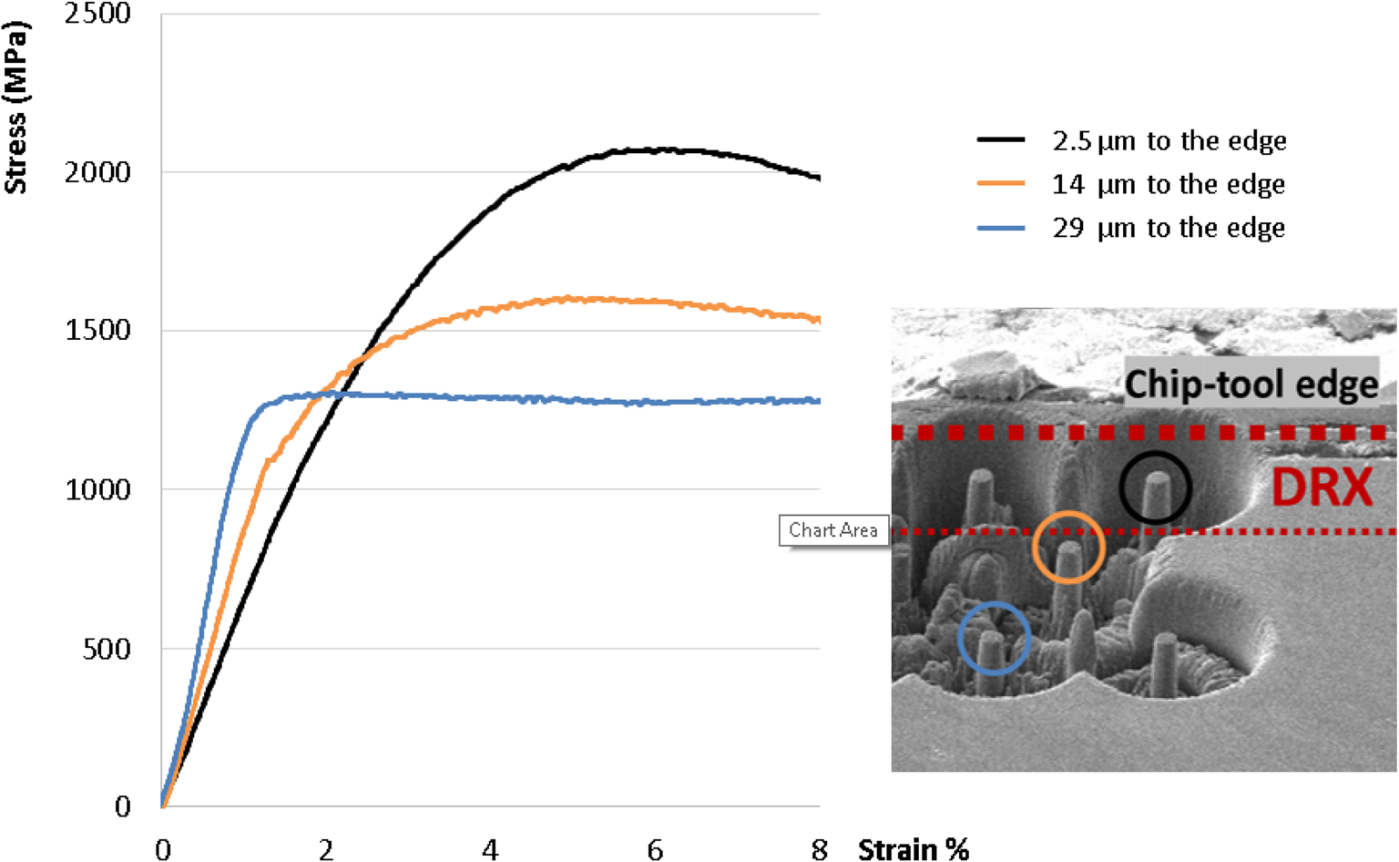
The graph shows the stress–strain plot for 2 µm diameter pillars milled at 2.5, 14 and 29 µm from the chip edge.

For the mechanical tests, pillars of 6, 3 and 2 µm in diameter have been milled by FIB on the cross-section at the very surface region and down to 63 µm deep into the sheared layer. The pillars heigh in each case is 3 times the diameter. For each case the pillar strength has been calculated at a strain of 5%. Figure 4 shows the values of strength depending on the distance of the center of the pillar to the edge. The pillar diameter did not make a notable influence on the strength, indicating no size-effect on the measurements. In contrast, the strength strongly depends on the distance to the surface, i.e. on the grain size and on the strain state of the material. Figure 5 shows individual stress–strain plots of 2 µm diameter pillars located at different distances from the surface.

**Figure 6:**
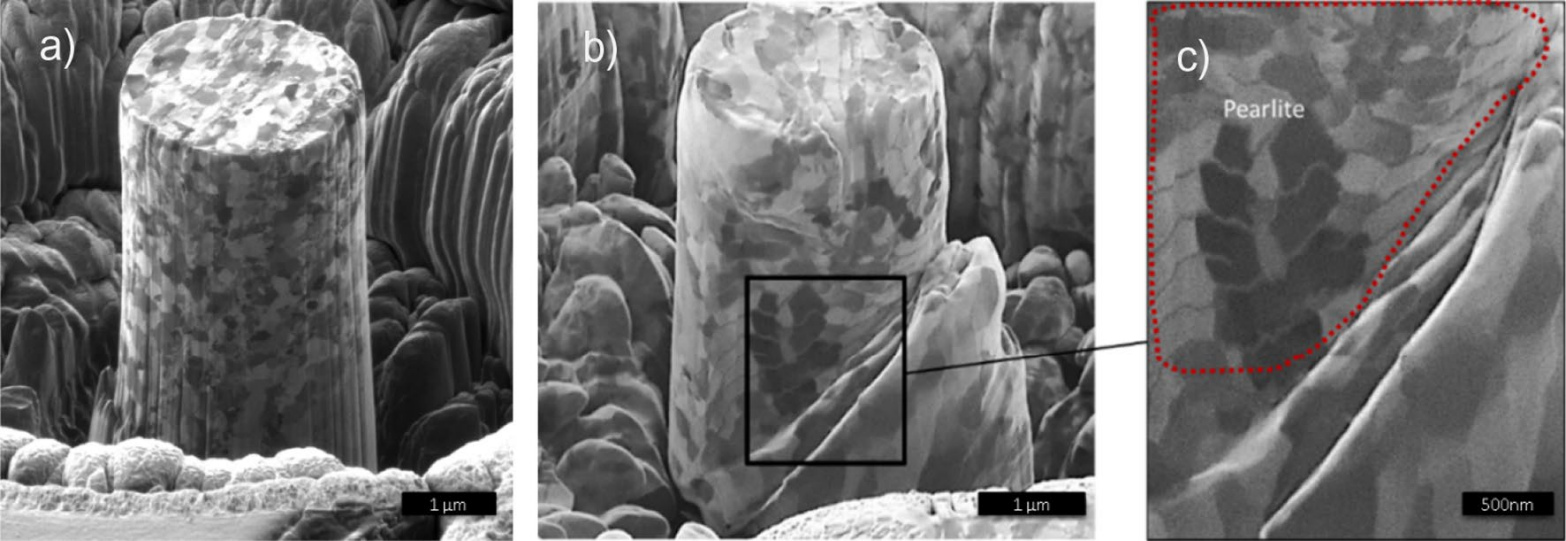
A pillar (FIB-scanning) image to reveal the grain distribution and the position of pearlite lamellae, locating the area of shear in the interface between ferrite and pearlite. Nanograined steel (**a**) depicts a homogeneous deformation, while in for larger grains the boundary of pearlite colonies (**b**) concentrates the shearing (**c**).

The curve representing the sheared layer (29 µm to the edge) demonstrates the maximum strength value of around 1300 MPa, while at the top of the DRX layer (2.5 µm to the edge), the maximum strength may exceed 2000 MPa. FIB images of compressed pillars (Fig. 6) reveal the difference in deformation behavior of the pillars: while the nanocrystalline pillar from the DRX layer shows a regular structure of nano-grains that deform homogeneously, the pillar from a depth of 29 µm contains the deformed pearlitic layers which provide an easy sliding plane.

**Figure 7:**
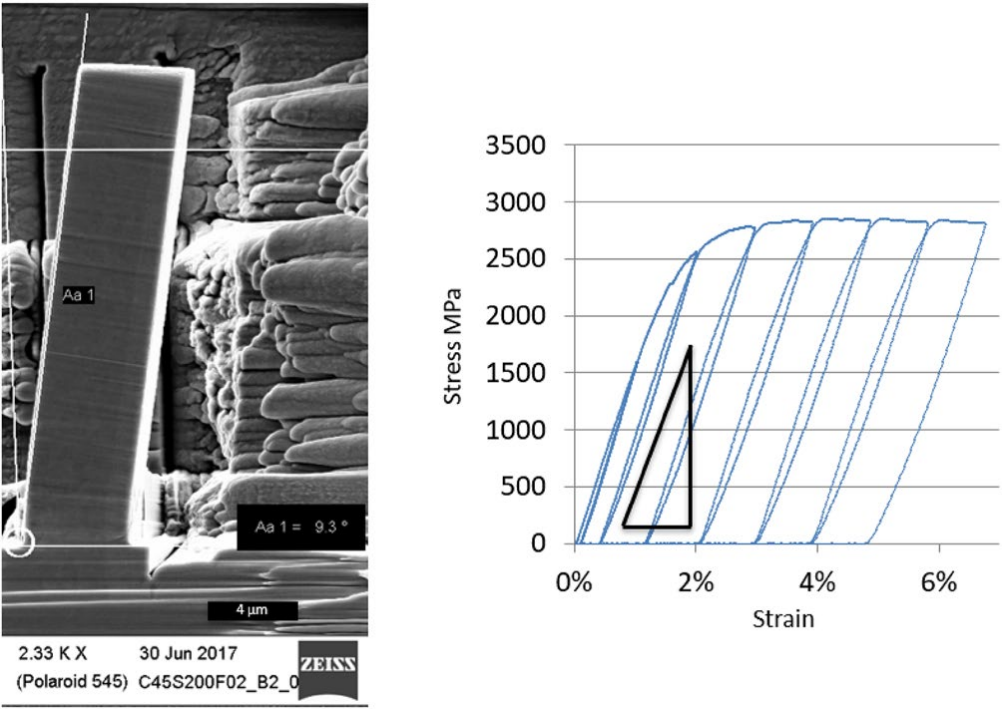
Beam bending experiments for elastic modulus calculation. Left: an SEM image of the bent beam after experiment; right: typical measured strain–stress curve for bending experiment.

One of the features observed in the curves of Fig. 5 is a slight reduction of the elastic modulus in the pillars in the recrystallized area. Variation of elastic modulus can indicate a fundamental change in atomic structure and/or interatomic distances^21^, thus pointing to some redistribution of the components in the lattice. However, determining the elastic modulus in micro-compression experiments does not give reproducible results, small misalignments lead to notable changes in the results. In order to obtain an accurate value of the elastic modulus beam bending experiments were performed in the proximity of the edge, inside the area affected by DRX. Two beams of 2 µm wide and two beams of 5 µm wide were bent in successive steps until an inclination of approximately 7 degrees. In each test between 4 and 6 cycles of load-unload were used to calculate the elastic modulus. The method of beam bending was previously demonstrated to be suitable for elastic modulus measurement at small scales^22^. It provides good sensitivity to modulus calculation and, contrary to pillar compression, it is relatively indifferent to small geometric misalignments. Beams with a width of 5 µm and 25 µm long have been tested by flexion in-situ in SEM (Fig. 7). Calculations of elastic modulus have been made following the procedure described by Demir et al.^23^. The set of beam bending tests has led to reproducible values of elastic modulus of around 178 GPa, with a standard deviation of 5 GPa.

## Discussion

In classical SDP methods for structure refinement, the achievable grain size, also known as the grain size of saturation, is defined by the competition between the rate of structure fragmentation and grain coarsening^24^: While sustained large strains break down the microstructure into smaller and more defective grains, high temperature activates grain boundary and lattice diffusion of atoms, the later drives diffusive repairing of crystallographic defects and coarsening of the grains^25^. These processes happen on relatively long timescales, which are unavoidable in bulk processing. The scenario of frictional surface structure refinement is slightly different. Though similar large strains are generated in a subsurface region upon friction, e.g. during machining, this happens very locally and on the timescale of milliseconds. Consequently, the area is rapidly cooled in approximately the same timeframe. As the activation energy of boundary diffusion is substantially lower than the one of lattice diffusion, the dominating process of structure relaxation in this case is rotational recrystallisation^8^ driven by boundary diffusion. Grain coarsening does not have a substantial impact in these short timeframes. This pushes the achievable grain size down to 100 nm and smaller. Fast cooling stabilizes the nano-structured layer, retaining this particular material assembly^12^. Grain size and residual internal strain distribution with depth below the surface reflect the distribution of the temperature field during the process and the time of exposition of the structure to high temperature. As both parameters have a strong dependence on the depth, the resulting structure also shows a peculiar depth dependence of the grain sizes and residual strain. The top layer, which is in direct contact with the tool, accommodates the highest temperature, but is cooled very fast. There strain remains over 300% in the few microns closest to the tool contact. Consequently, highly strained material in this region relaxes (by rotational DRX) into sub 100 nm strain-free grains, which have no time to grow. 5–10 µm deeper into the bulk, high temperatures remain for a longer time and thus larger grains are observed. At 20 µm depth the elevation of the temperature during processing and strains under 200% are not sufficient to initiate diffusion and we observe highly strained material in this region. Figure 6 demonstrates that pearlitic layers are preserved here yet are strongly deformed.

On top of structural refinement, this process in pearlitic steel also results in redistribution of carbon. In pristine material pearlite colonies occupy 3/4 of the volume, while in the DRX layer we do not see cementite lamellae or grains, and only a few precipitates of other steel elements (Fig. 2). This lack of carbides might indicate a scenario where carbon atoms diffuse to grain boundaries and decorate them, but chemical analysis in Fig. 2 does not show an excess of carbon at the grain boundaries. Carbon is mostly homogeneously distributed in the matrix of ferrite, confirming the results of a number of authors reporting a super-saturated state of ferrite after cold working of pearlitic steel caused by severe plastic deformation^26–28^. Carbon dissolution in ferrite above the saturation level has been unambiguously experimentally validated by atom probe tomography^29^ on machined surfaces of martensitic steels. This supersaturated ferrite does not reproduce the structure of other metastable carbon-rich iron structures, like martensite or austenite. On the contrary, it possesses a relaxed ferrite structure with high carbon content and without carbon segregation. The effects of strain-induced cementite decomposition have been seen in other processes where a large strain is applied to pearlitic steels, like in wire drawing^30^. It has been proposed that the decomposition occurs by progressive thinning of pearlite lamelae^31^, which is favored by misfits between the ferrite and cementite lattices^32^. It has been proposed that when the carbon atoms reach the iron lattice, they accommodate and move following screw dislocations, predominantly generated in large strain processes^28^. This mechanism of carbon-dislocation interaction would strongly increase the Peierls stress contributing to the remarkable mechanical properties observed in supersaturated ferrite.

Mechanical properties of the observed FIN layers were characterized by micromechanical testing methods: micropillar compression and micro-beam bending. Usage of micro-scale methods allows the spatial distribution of properties to be obtained from a ~ 30 µm layer.

Experiments in pillar compression were designed to account for possible size effects, i.e. the dependence of the measurements on the pillar diameter due to dislocation starvation^33,34^, which typically cause an increase in measured strength in single crystals. The grains in the FIN layer are well below 1 µm and Fig. 4 clearly shows that there is no dependence of the measured strength on the pillar diameter, meaning we are measuring a real material strength as expected when the grains size is much smaller than the pillar diameter.

The strength of the nanostructured material in the DRX layer is almost 3 times higher than that of the original AISI1045 steel—~ 2000 MPa against 500–700 MPa in a pristine AISI1045. This is a typical trend in nanostructured metals, though here it is measured for the first time in high carbon content steel. Figure 6 shows that the deformation and failure mechanisms in nanostructured material and in distorted pearlite may be substantially different. While pearlitic layers inclined to the strain direction provide “easy” sliding planes (Fig. 6), in the isotropic nanostructured material from the DRX layer there is no preferential “easy” direction, which in combination with the high density of the grain boundaries (serving as the sinks for dislocations) inhibits sliding and determines enhanced strength.

The other trend visible in Fig. 5 is the notable decrease of the slope of the elastic part of the strain/stress curves (elastic modulus) at increasing ultimate strength of the pillar. This qualitative observation on the pillar compression has been quantitatively evaluated in beam bending experiments (Fig. 7), the measurements resulted in a 178 ± 5 GPa elastic modulus for the DRX layer, which is 12% less than the elastic modulus of pristine AISI1045. A decrease in elastic modulus by about 7% after rolling or tensile tests has been reported previously^1,35^, linking metal defect generation with elastic modulus. Chen et al. have found a reduction of the elastic modulus in the order of 10% in the “white” (DRX) layer in friction processed Inconel 718^36^. This effect has also been observed in other metals with crystallite sizes below 25 nm^37^, where the volume fraction of defects may infer certain characteristics of glasses, in particular the reduced elastic modulus^38^. Thus, the same process of structure refinement that makes the strength higher also brings down the elastic modulus.

In summary:

- FIN layer is generated from AISI1045 during machining, which is characterized by the gradient of crystal size and internal stress.
- The topmost 5 µm of the FIN layer undergoes rotational DRX and fast cooling, which preserves the crystal size at the level of 100 nm.
- Carbon distributes uniformly in the ferrite lattice.
- Recrystallized layer has a strength of 2000 MPa, which is three times over the typical value of pristine AISI 1045 steel.
- Elastic modulus of nanostructured layer is reduced by 12% relative to pristine AISI1045.

## Methods

Chips generated during high-speed orthogonal dry cutting of AISI 1045 annealed steel were used as representative samples of friction induced nanostructures. Dry machining was performed with uncoated P15 grade carbide cutting tools (WIDIA TPUN 160308 TTM), rake angle = 6 degrees and cutting-edge radius = 40 µm. The feed rate was kept constant to 0.2 mm/rev, while the cutting speed was 200 m/min. At these conditions the pristine material (a mean grain size of 7–9 µm, and microstructure of pearlite and ferrite) undergo severe plastic deformation (over 200%) accompanied by high temperature (800 ºC) and consequent fast cooling (approximately within 3 ms)^39^. High temperature initiates dynamic crystallization on the surface of severely deformed material and fast cooling freezes the obtained nanocrystallites preventing grain coarsening^8^.

Chips were embedded in silver epoxy, polished and used for EBSD analysis. The same samples were utilized for fabrication and testing of micro-pillars. These pillars were made by FIB milling in two steps. First, a rough milling was performed with currents up to 9 nA. Subsequently, each pillar was exactly defined using ion current of 100 pA. Beams for bending tests were milled at the edges of the chips side polished without embedding; this was necessary to have access for milling from two sides. Beams of 2 and 5 µm thickness have been milled by FIB with first a high current (9 nA), and afterwards a fine milling was performed at 1 nA. All the FIB nanofabrication was made on a DualBeam SEM/FIB instrument (Helios 600 DualBeam, FEI). The STEM study was conducted on a Titan 60–300 (FEI) equipped with an EDX RTEM detector (EDAX). The EDX spectrum images were decomposed by Minimum Linear Least Square (MLLS) method resulting in the elemental maps depicted in Fig. 2. (t-)EBSD data were obtained on a Helios 600 DualBeam equipped with AMETEK EBSD camera and on a Sigma SEM (Zeiss) equipped with an Oxford Instruments EBSD camera.

**Figure 8:**
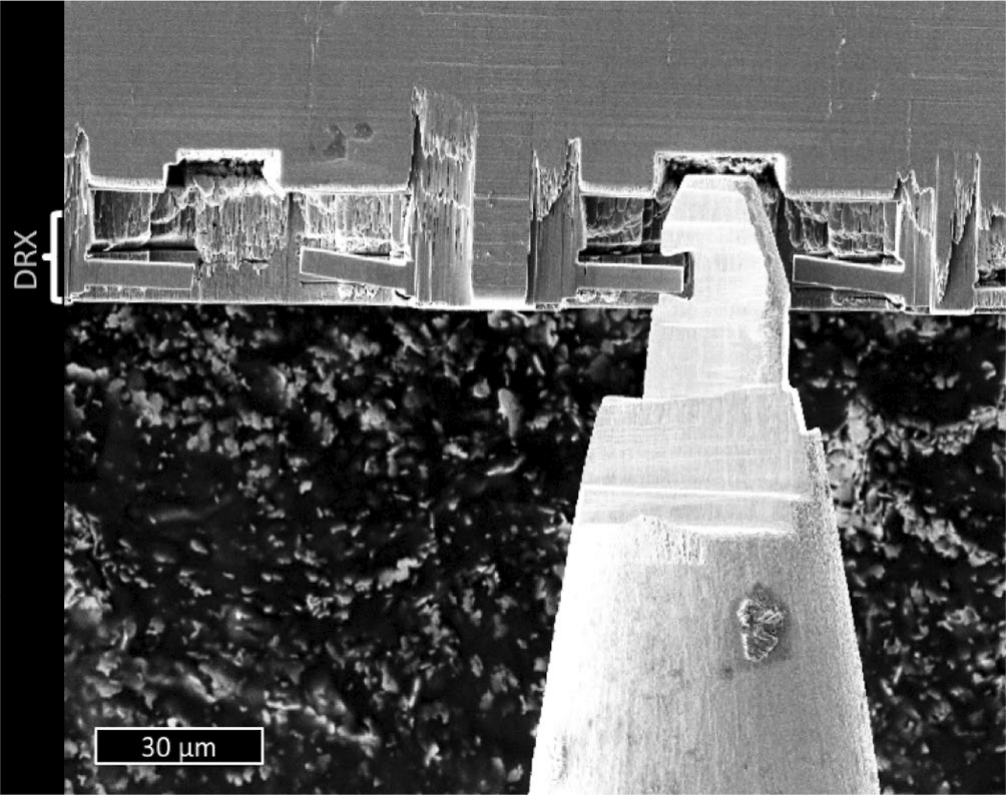
Set-up for beam bending in-situ in the SEM experiments. Tip has been fabricated by FIB milling of a tungsten needle.

Pillar compression tests were made using a nano-indenter TriboIndenter TI 900 (Hysitron) equipped with a flat punch tip. Pillars have been compressed by displacement control at a strain rate of approximately 40 s^−1^. Sneddon criteria was used for the correction of the base material deformation (Sneddon 1965). Experiments of beam bending were made in-situ in the chamber of the Sigma SEM microscope (Zeiss). The testing device in that case was nano-indenter UNAT-SEM2 (ASMEC). This in-situ nano-indenter was equipped with a tungsten tip milled by FIB to perform bending tests as in Fig. 8.

**Figure 9:**
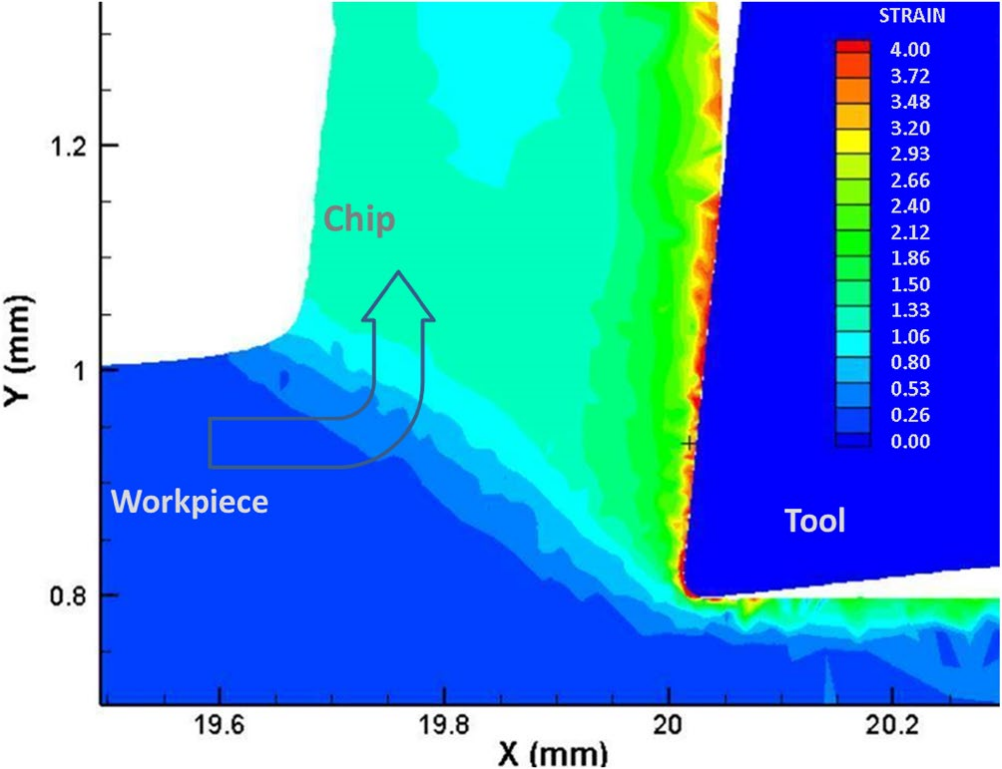
Calculations of the strain in workpiece and chip for a AISI 1045 steel cut at 200 m/min and uncut chip thickness of 0.2 mm.

Software assisted modelling was used to calculated to plastic strain of the material. The plastic strain values in the tool-chip contact area are higher than 300% considering results obtained with AdvantEdge (V7.9) commercial software (Fig. 9). Material and contact data employed in the simulation were taken from the software database. Cutting conditions, tool geometry and material were similar to those employed in experimental tests.

## Data Availability

The datasets used and/or analysed during the current study available from the corresponding author on reasonable request.
